# Three Efficacy of *Ligilactobacillus plantarum* WB21 Against Halitosis Induced by *Porphyromonas gingivalis*

**DOI:** 10.3390/microorganisms14061283

**Published:** 2026-06-06

**Authors:** Hyon-Mo Ku, Sung-Hoon Lee

**Affiliations:** 1 Oral Health Center for People with Disabilities, Dankook University Dental Hospital, Cheonan 31116, Republic of Korea; dku.kuhyonmo@dankook.ac.kr; 2 Department of Oral Microbiology and Immunology, College of Dentistry, Dankook University, Cheonan 31116, Republic of Korea

**Keywords:** halitosis, volatile sulfur compounds, *Ligilactobacillus salivarius*, preventive effect, *Porphyromonas gingivalis*

## Abstract

Halitosis is caused by volatile sulfur compounds (VSCs) produced by periodontopathogens. The aim of this study is to examine the mechanism by which *Ligilactobacillus salivarius* WB21 inhibits halitosis. A susceptibility assay for *Porphyromonas gingivalis* was conducted using spent culture media (SCM) from *L. salivarius* WB21, and VSCs from the suspension of *P. gingivalis* were analyzed in the presence or absence of the SCM. After co-cultivating *P. gingivalis* and *L. salivarius*, *P. gingivalis* growth and VSC levels were measured using a spectrophotometer and a gas chromatograph, respectively. Additionally, levels of methyl mercaptan in the suspension and in the *mgl* gene of *P. gingivalis* were investigated. The SCM from *L. salivarius* WB21 significantly inhibited growth of *P. gingivalis* (*p* < 0.05) and significantly reduced emission of VSCs from the suspension of *P. gingivalis* (*p* < 0.05). When *L. salivarius* WB21 was present in a co-culture condition, *P. gingivalis* growth was significantly inhibited, and levels of methyl mercaptan in the culture medium were also reduced (*p* < 0.05). Finally, expression of the *mgl* gene of *P. gingivalis* was significantly reduced under co-cultivation with *L. salivarius* WB21 (*p* < 0.05). *L. salivarius* WB21 may inhibit colonization of periodontopathogens in the oral cavity and suppress production and emission of VSCs. Therefore, *L. salivarius* WB21 may be effective in treating halitosis when applied to the oral cavity.

## 1. Introduction

Bad breath has recently become a problem in social life. It is known as halitosis, oral malodor, or fetor oris [1]. Among these terms, “halitosis” is used professionally in dentistry. Temporary halitosis emanates from the digestive tract after consumption of certain foods or drinks [2]. Persistent halitosis, however, mainly occurs due to metabolites known as volatile sulfur compounds (VSCs) produced by Gram-negative anaerobic bacteria in the oral cavity [3]. These VSCs include hydrogen sulfide (H_2_S), methyl mercaptan (CH_3_SH), and dimethyl sulfide (CH_3_SCH_3_), and their levels are used as an indicator of halitosis [1].

Gram-negative anaerobes related to halitosis include *Porphyromonas gingivalis*, *Tannerella forsythia*, and *Treponema denticola* [3]. These bacteria, classified as the red complex, are known to cause periodontitis. They are asaccharolytic microbes. Another characteristic of these bacteria is that they are benzoyl-DL-arginine-naphthylamide (BANA)-positive, meaning that they exhibit a high level of proteolytic activity through a trypsin-like enzyme [3,4]. Specificially, *P. gingivalis* has L-methionine-α-deamino-γ-mercaptomethane-lyase (encoded by *mgl* gene), which can produce a large amount of methyl mercaptan (CH_3_SH) from methionine and cysteine and is more frequently detected in halitosis patients [5].

*Ligilactobacillus salivarius*, formerly named *Lactobacillus salivarius*, was initially introduced by Rogosa in 1953 [6]. This bacterium is considered a safe strain by the European Food Safety Authority (EFSA) and the United States Food and Drug Administration (FDA) [7,8]. Therefore, many subtypes have been used as probiotics. This probiotic bacterium is known to produce antibacterial peptides called bacteriocins which serve as tools for competing against other bacteria in the environment [9]. The bacteriocins of *L. salivarius* are classified as class II non-lanthionine-containing peptides; representative examples include Abp118, *salivaricin* T, *salivaricin* L and *salivaricin* P [10]. In addition, *L. salivarius* produces active metabolites, such as exopolysaccharides, organic acids, and short-chain fatty acids [6], which have been proven to have anti-inflammatory, antioxidant and immune regulating properties [11,12,13]. Research has shown that *L. salivarius* may have potential beneficial effects on human health.

Halitosis is a common problem that can be caused by periodontitis, poor oral hygiene, or tongue debris [14]. Ultimately, halitosis is attributed to dysbiosis of the oral microbiome and an increasing proportion of periodontopathogens. In particular, *P. gingivalis* is recognized as the primary causative bacterium for this condition [15]. Various efforts have recently been undertaken to utilize probiotics to address the conditions that lead to halitosis, and their effectiveness has been assessed through clinical trials. However, the specific mechanisms by which they exert their effects on halitosis have not been clearly elucidated.

While probiotics have recently emerged as a potential intervention for halitosis, their efficacy remains strain-specific due to variations in microbial metabolism and metabolite production. Consequently, the inhibitory impact of probiotics on halitosis is not uniform. Therefore, the present study aims to elucidate the specific mechanisms by which *L. salivarius* WB21 inhibits *P. gingivalis* activity as it relates to halitosis.

## 2. Materials and Methods

### 2.1. Bacterial Strain and Culture Condition

*Ligilactobacillus salivarius* WB21 was maintained in Man, Rogosa and Sharpe (MRS) broth (BD Biosciences, Franklin Lakes, NJ, USA) at 37 °C under aerobic condition. The strain, deposited at the National Institute of Technology and Evaluation (Japan) under the accession number FERM BP-7792, was provided by Ju Yeong NS Co., Ltd. (Seoul, Republic of Korea). In order to investigate antimicrobial activity and neutralizing efficacy, the WB21 strain was cultivated into tryptic soy broth (TSB) (BD biosciences). *Porphyromonas gingivalis* ATCC 33277 was used to generate volatile sulfur compounds (VSCs); this was cultured using TSB supplemented with hemin (1 μg/mL; Sigma-Aldrich Co., St Louis, MO, USA) and vitamin K (0.2 μg/mL; Sigma-Aldrich) at 37 °C under anaerobic condition (5% H_2_, 10% CO_2_, and 85% N_2_).

### 2.2. Antibacterial Activity

To evaluate bacteriocin production by *L. salivarius*, the susceptibility of *P. gingivalis* to conditioned media derived from *L. salivarius* was assessed. A susceptibility assay was carried out according to Clinical Laboratory Standard Institute (CLSI) guidelines. First, spent culture medium (SCM) of *L. salivarius* was collected using a polyvinylidene fluoride (PVDF) filter (0.22 μm pore size). The cultured *P. gingivalis* was counted using a bacterial counting chamber (Marienfeld Superior, Lauda-Königshofen, Germany) and adjusted to 1.5 × 10^7^ cells/mL with fresh TSB medium. Next, 180 μL of TSB was dispensed into rows B through G of a 96-well plate well (SPL Life sciences, Pocheon, Republic of Korea). The prepared SCM (180 μL) was added to the first column of rows C, E, and G, followed by 2-fold serial dilution up to the 11th column. The adjusted *P. gingivalis* suspension (20 μL) was then inoculated into the prepared 96-well plate. The plate was incubated at 37 °C under anaerobic condition for 36 h, and bacterial growth was measured by optical density at a wavelength of 660 nm using a microplate reader (BioTek, Winooski, VT, USA).

### 2.3. Co-Cultivation

To investigate the co-existence characteristics of *P. gingivalis* and *L. salivarius* WB21 in the oral cavity, the WB 21 strain was co-cultured with *P. gingivalis* using cell culture inserts (SPL life Sciences, Pocheon, Republic of Korea). The cultured *P. gingivalis* and *L. salivarius* WB21 were counted using a bacterial counting chamber (Marienfeld Superior) and adjusted to 1.0 × 10^7^ and 1.0 × 10^8^ cells/mL, respectively. The *P. gingivalis* suspension was inoculated into the upper chamber (inside) while the *L. salivarius* suspension was inoculated into the lower well (outside) of the insert at volumes 1, 5, and 10 times that of *P. gingivalis,* in line with the manufacturer’s recommended volumes. The plate was incubated at 37 °C under anaerobic atmosphere for 36 h. Growth of *P. gingivalis* was then measured by optical density at a wavelength of 660 nm using a spectrophotometer (BioTek).

### 2.4. Measurement of VSCs

The SCMs of *P. gingivalis* and *L. salivarius* were collected. The *P. gingivalis* SCM was dispensed into a 50 mL conical tube, to which various volumes of *L. salivarius* SCM were added. The *P. gingivalis* SCM alone (as a control) and the resulting mixtures were mixed using a rotator (IKA, Staufen, Germany) for 1 min. Subsequently, headspace gas was collected using a 1 mL syringe (BD biosciences, Franklin Lakes, NJ, USA) from just above the medium surface. VSC levels were measured using an Oral Chroma^TM^ gas chromatograph (FIS Inc., Itami Hyogo, Japan). In a separate experiment, suspensions of *P. gingivalis* from either single cultures or co-cultures with the WB21 strain were transferred into 50 mL conical tubes. The tubes were rotated on a rotator, and headspace gas was immediately collected using a 1 mL syringe above the suspension surface. The VSCs were measured using the Oral Chroma^TM^ gas chromatograph.

### 2.5. Methyl Mercaptan Assay

The production of methyl mercaptan (CH_3_SH) from *P. gingivalis* was measured using 5,5′-dithiobis (2-nitrobenzoic acid) (DTNB; Sigma-Aldrich Co., St Louis, MO, USA). Briefly, *P. gingivalis* was either single- or co-cultured with *L. salivarius* WB21 in cell culture inserts for 36 h. Afterwards, 50 μL of *P. gingivalis* suspension was transferred into the wells in 96-well plate. Then, 50 μL each of L-methionine (0.6%, *w*/*v*) and DTNB (0.06%, *w*/*v*) was added to each well. The plate was incubated for 10 h. Production of methyl mercaptan was then measured at a wavelength of 430 nm using a spectrophotometer.

### 2.6. Analysis of Mgl Gene Expression

The effects of *L. salivarius* WB21 on expression of methionine gamma-lyase (*mgl*), an enzyme associated with VSC production in *P. gingivalis*, were investigated at the transcript level. *P. gingivalis* and *L. salivarius* WB21 were co-cultivated in cell culture inserts at ratios of 1:1 and 1:5 for 10 h using the aforementioned co-cultivation method. *P. gingivalis* was collected by centrifugation at 5000× *g* at 4 °C, and total RNA was immediately extracted using TRIzol^TM^ reagent (Invitrogen, Waltham, MA, USA). cDNA was synthesized from 1 μg of total RNA using Maxine RT-premix with Random primer (iNtRON, Seongnam, Republic of Korea). cDNAs were mixed with 10 μL of TB green Premix Ex Taq (Takara Co., Kyoto, Japan) and 0.4 μM of each primer pair in a final volume of 20 μL. Quantitative PCR was performed for 40 cycles (95 °C for 15 s, 60 °C for 15 s, and 72 °C for 33 s) using an ABI 7500 Real-time PCR system (Applied Biosystems, Foster City, CA, USA). The specificity of the PCR products was verified by melting curve analysis. 16S rRNA was used as a housekeeping gene to normalize expression levels and to quantify changes in *mgl* expression between single- and co-cultured *P. gingivalis*. The primer sequences for Real-Time PCR were as follows: forward 5′-TTC CGA GCT TCC CCC AAT AC-3′ and reverse 5′-ATG AGG GTT TCC GTA TCG CC-3′ for the *mgl* gene; forward 5′-TGT AGA TGA CTG ATG GTG AAA ACC-3′ and reverse 5′-TTT AGA GAT TCG CAT CCG GT-3′ for the 16S rRNA gene.

### 2.7. Statistical Analysis

Experiments were performed in triplicate. Statistical analysis was conducted using IBM SPSS statistics (version 23; IBM, Armonk, NY, USA). Data were assessed for normal distribution using the Kolmogorov–Smirnov test. As the data were not normally distributed, data from the different groups were analyzed by using the non-parametric Kruskal–Wallis test and post hoc analysis. Comparison of differences between individual groups was carried out using the Mann–Whitney U test. Differences with a *p*-value of less than 0.05 were considered statistically significant.

## 3. Results

### 3.1. Antibacterial Activity Against P. gingivalis

**Figure 1:**
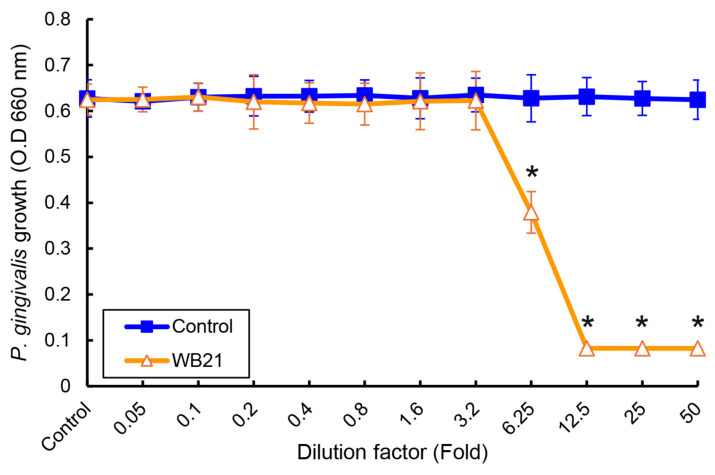
Inhibition of *P. gingivalis* growth by spent culture medium from *L. salivarius* WB21. *L. salivarius* WB21 was cultivated using MRS broth, and the spent culture medium (SCM) was then collected. A susceptibility assay of *P. gingivalis* for the SCM was evaluated. An asterisk (*) indicates a significant difference compared to the control group (*p* < 0.05).

**Figure 2:**
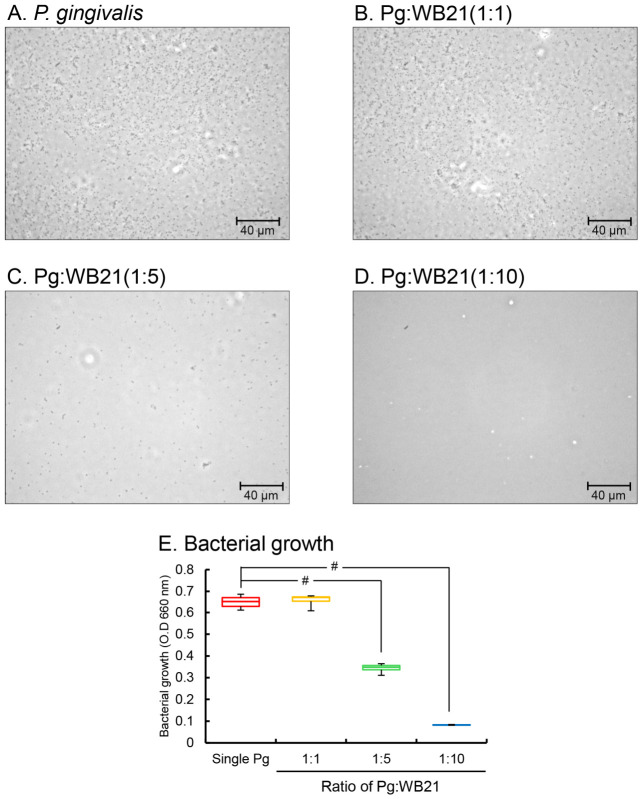
Inhibitory effect of *L. salivarius* WB21 on growth of *P. gingivalis* in a co-cultivating condition. *P. gingivalis* was co-cultivated with or without *L. salivarius* WB21 using a cell culture insert. The suspension of *P. gingivalis* was observed using a microscope (**A**–**D**), and *P. gingivalis* growth was measured using a spectrophotometer (**E**). A sharp (#) indicates a significant difference compared to single-cultured *P. gingivalis* (without *L. salivarius* WB21) (*p* < 0.05).

To examine the antibacterial activity of *L. salivarius* WB21 against *P. gingivalis*, the SCM of *L. salivarius* WB21 was collected and tested. According to a susceptibility assay based on CLSI guidelines, the WB21 strain exhibited antibacterial activity against *P. gingivalis* (Figure 1). Growth of *P. gingivalis* was significantly inhibited at SCM concentrations of 6.25% or higher (*p* < 0.05). Furthermore, the WB21 strain inhibited *P. gingivalis* growth within the co-culture system (Figure 2). The abundance of *P. gingivalis* was significantly decreased when inoculated with *L. salivarius* WB21 at a ratio of 1:5 or higher (*p* < 0.01).

### 3.2. Neutralizing Effect of WB21 SCM on the Emission of VSCs Produced by P. gingivalis

**Figure 3:**
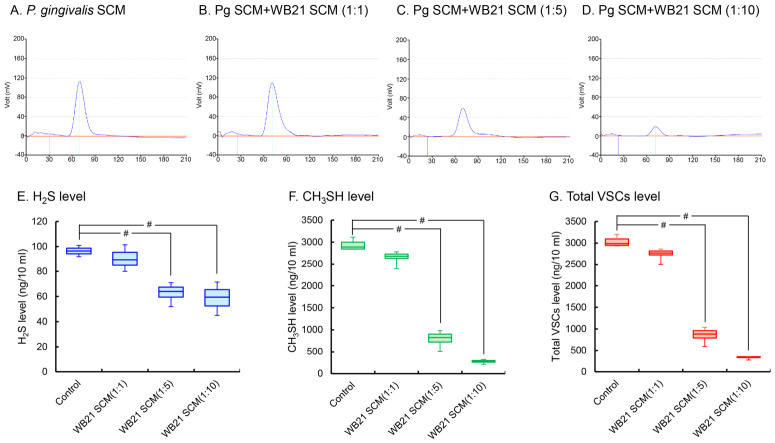
Inhibitory effect of *L. salivarius* WB21 SCM on emission of VSCs. *P. gingivalis* was cultured with BHI broth, and the suspension of *P. gingivalis* was mixed with *L. salivarius* SCM in various ratios. After collecting the gas above the mixture, VSCs were analyzed using a gas chromatograph. Total VSCs were expressed as peaks (**A**–**D**). Levels of H_2_S (**E**), CH_3_SH (**F**), and total VSCs (**G**) were expressed as a box plot. A sharp (#) indicates a significant difference compared to the control group (*p* < 0.05).

The SCMs from *L. salivarius* and *P. gingivalis* were mixed, and gaseous VSC levels were measured using a gas chromatograph. Total gaseous VSC levels were significantly reduced at an SCM mixture with a WB21 ratio of 1:5 or higher (Figure 3C,G) (*p* < 0.01). Furthermore, concentrations of hydrogen sulfide (H_2_S) and methyl mercaptan (CH_3_SH) were also significantly decreased in the mixture containing *P. gingivalis* SCM and WB21 SCM at ratios of 1:5 or above (Figure 3E,F).

### 3.3. Reducing Effect of L. salivarius WB21 on VSCs Produced by P. gingivalis

**Figure 4:**
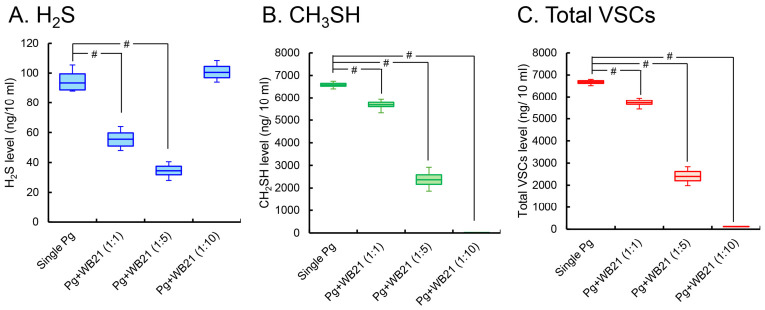
Inhibitory effect of *L. salivarius* WB21 on production of VSCs from *P. gingivalis* in co-cultivating condition. *P. gingivalis* was co-cultivated with or without *L. salivarius* WB21 using a cell culture insert. VSCs from the suspension of *P. gingivalis* was analyzed using a gas chromatograph. A sharp (#) indicates a significant difference compared to single-cultured *P. gingivalis* (*p* < 0.05).

To investigate VSC production by *P. gingivalis* when co-existing with *L. salivarius* WB21, gaseous VSCs released from *P. gingivalis* suspensions were measured. Total VSCs and methyl mercaptan levels were significantly reduced in the *P. gingivalis* suspension when co-cultured with the WB 21 strain at a ratio of 1:1 or above (Figure 4B,C) (*p* < 0.05). Interestingly, hydrogen sulfide levels were significantly decreased in the suspensions from co-cultures with the WB 21 strain at ratios of 1:1 and 1:5 (Figure 4A) (*p* < 0.05).

### 3.4. Inhibition of Methyl Mercaptan Produced by P. gingivalis

**Figure 5:**
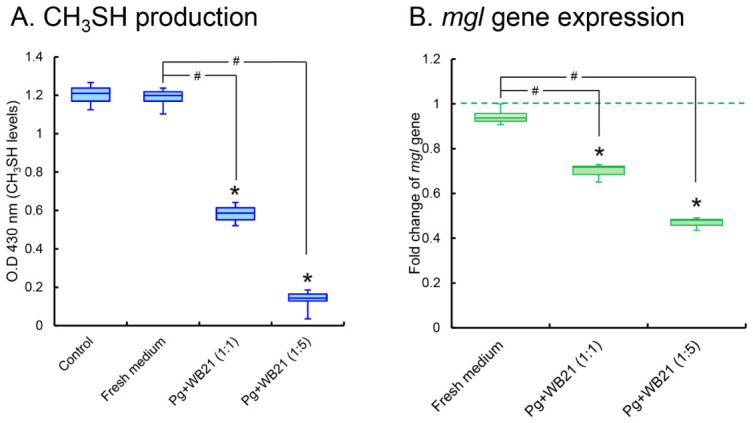
Suppression effect of *L. salivarius* WB21 on production of methyl mercaptan. *P. gingivalis* was co-cultivated with *L. salivarius* WB21 in various concentrations, and the conditioned media and bacteria were then collected separately from the *P. gingivalis* suspension. Levels of methyl mercaptan (CH_3_SH) were measured in the conditioned media (**A**); then, after total RNA was extracted from the bacteria, *mgl* expression was investigated using real-time RT-PCR (**B**). The dot line is the level of the control group. An asterisk (*) indicates a significant difference compared to the control group (*p* < 0.05), and a sharp (#) indicates a significant difference compared to the fresh medium group (*p* < 0.05).

To evaluate whether the reduction in gaseous VSC levels in the co-culture system was due to suppression of gas emissions or to a decrease in VSC production by *P. gingivalis*, the levels of methyl mercaptan in the SCM were measured, and expression of the associated gene was examined. The methyl mercaptan levels in the *P. gingivalis* suspension were significantly lower in the groups co-cultured with *L. salivarius* WB21 strain at 1:1 and 1:5 ratios, compared to the MRS fresh medium (Figure 5A) (*p* < 0.05). As methyl mercaptan was produced by the methionine gamma-lyase of *P. gingivalis*, *mgl* gene expression was evaluated in the presence of *L. salivarius* WB21. The expression level of the *mgl* gene in single-cultured *P. gingivalis* was set to 1 for comparison with its expression during co-cultivation with *L. salivarius* WB21. In the absence of the WB21 strain (control), *mgl* expression did not show a significant difference (Figure 5B). However, *mgl* gene expression was significantly reduced in the groups inoculated with the WB21 strain at 1:1 and 1:5 ratios (*p* < 0.01).

## 4. Discussion

Halitosis increasingly disrupts daily life. This has led to exploration of various therapeutic approaches; these include the use of probiotics, which have recently emerged. Because different probiotic bacteria have distinct metabolic processes and produce different metabolites, their inhibitory effects on halitosis may also vary. In the present study, the inhibitory effects of *L. salivarius* WB21 on factors related to halitosis were examined.

It is known that halitosis is caused by periodontitis-related bacteria [16] which increase production of volatile sulfur compounds (VSCs). Therefore, the antibacterial activity of *L. salivarius* WB21 against *P. gingivalis* was evaluated, and the SCM of *L. salivarius* WB21 was found to inhibit growth of *P. gingivalis*. Furthermore, in co-culture, growth of *P. gingivalis* was significantly inhibited by *L. salivarius* WB21. These results suggest that oral administration of *L. salivarius* WB21 could limit *P. gingivalis* colonization, potentially reducing VSC concentrations and mitigating halitosis. Additionally, beyond *P. gingivalis*, *L. salivarius* WB21 has been shown to demonstrate antibacterial activity against other key periodontal pathogens, including *Tannerella forsythia* and *Treponema denticola* [17].

Next, the effects of *L. salivarius* WB21 on emission of VSCs were examined using SCMs. When the SCMs of *L. salivarius* and *P. gingivalis* were mixed in various ratios, gaseous VSCs decreased in a dose-dependent manner relative to the quantity of *L. salivarius* SCM. Furthermore, in the co-culture system, *L. salivarius* WB21 suppressed VSC production of *P. gingivalis* even at ratios that did not inhibit the growth of *P. gingivalis*. *P. gingivalis* is an asaccharolytic bacterium that metabolizes peptides exclusively to obtain energy [18]. Consequently, to digest these peptides, it secretes enzymes which in turn produce VSCs. Although some probiotics have been reported to bind to VSCs in the bodies of bacteria and inhibit their emission, *L. salivarius* WB21 did not show such an effect [1]. This was confirmed by mixing the SCM of *P. gingivalis* with varying concentrations of whole *L. salivarius* WB21 cells and incubating the mixture for 5 min. When gaseous VSCs were measured using gas chromatography, no significant differences were observed across all groups.

Finally, we evaluated whether *L. salivarius* WB21 only inhibits the emission of VSCs in a suspension or it suppresses VSCs production by inhibiting the enzymes secreted by *P. gingivalis*. L. *salivarius* WB21 was found to reduce methyl mercaptan levels in the *P. gingivalis* SCM and decrease the expression of the *mgl* gene. The reduction in methyl mercaptan levels in the SCM suggests that *L. salivarius* WB21 decreases the level of VSC-producing enzymes in *P. gingivalis*, thereby not only inhibiting VSC emission but also reducing VSC production. L-methionine-α-deamino-γ-mercaptomethane-lyase (METase), which is encoded by the *mgl* gene, produces methyl mercaptan (CH_3_SH), ammonia, and α-ketobytyrate from methionine and cysteine [5]. The correlation between *mgl* expression and CH_3_SH production has been reported in previous studies where a decrease in *mgl* expression resulted in the inhibition of CH_3_SH production [5,19].

One of the most important health-related attributes of probiotics is the production of antimicrobial substances, and extensive research is focused on the properties of bacteriocins. Bacteriocins produced by lactic acid bacteria are categorized based on the presence of lanthionine into lanthionine-containing bacteriocins (LCBs) and non-lanthionine-containing bacteriocins (NLCBs) [20]. The bacteriocin produced by *L. salivarius* is an NLCB, as it is a bacterium that produces bacteriocin without lanthionine. LCB-producing bacteria such as *L. plantarum*, *L. casei*, and *L. delbrueckii* possess cystathionine γ-lyase, which is encoded by the *met*C gene [21]. This enzyme produces VSCs from methionine; however, *L. salivarius* lacks the *met*C gene and therefore does not produce VSCs.

In conclusion, *L. salivarius* WB21 exhibits potent antimicrobial activity against *P. gingivalis* through the production of non-lanthionine-containing bacteriocins. Furthermore, *L. salivarius* WB21 was shown to reduce both emission and production of VSCs by *P. gingivalis*. These findings suggest that *L. salivarius* WB21 may effectively inhibit colonization by periodontopathogens in the oral cavity while suppressing the synthesis and release of VSCs. Consequently, the application of *L. salivarius* WB21 may be effective in treating halitosis.

## Data Availability

The original contributions presented in this study are included in the article. Further inquiries can be directed to the corresponding author.
