# Correction: Ku, H.-M.; Lee, S.-H. Three Efficacies of *Ligilactobacillus salivarius* WB21 Against Halitosis Induced by *Porphyromonas gingivalis*. *Microorganisms* 2026, *14*, 1283

**DOI:** 10.3390/microorganisms14102339

**Published:** 2026-10-14

**Authors:** Hyon-Mo Ku, Sung-Hoon Lee

**Affiliations:** 1 Oral Health Center for People with Disabilities, Dankook University Dental Hospital, Cheonan 31116, Republic of Korea; 2 Department of Oral Microbiology and Immunology, College of Dentistry, Dankook University, Cheonan 31116, Republic of Korea

In the original publication [1], there was a mistake in the title as published. The corrected title is as follows: “Three Efficacies of *Ligilactobacillus salivarius* WB21 Against Halitosis Induced by *Porphyromonas gingivalis*”. The authors state that the scientific conclusions are unaffected. This correction was approved by the Academic Editor. The original publication has also been updated.
